# Scientific Twitter: The flow of paleontological communication across a topic network

**DOI:** 10.1371/journal.pone.0219688

**Published:** 2019-07-30

**Authors:** Richard T. Bex, Lisa Lundgren, Kent J. Crippen

**Affiliations:** School of Teaching and Learning, College of Education, University of Florida, Gainesville, Florida, United States of America; Beihang University, CHINA

## Abstract

The field of paleontology, which is based principally on observations of the natural world, includes an active community that is engaged across multiple social media platforms, consisting of museums, academic researchers, amateur fossil collectors, paleontological artists, and commercial fossil dealers. As such, it represents an ideal environment for examining the people, interactions, and flow of scientific information. Using interactions involving the four most popular Twitter hashtags for paleontology, this embedded mixed methods study defined the members of this social world and investigated how they influenced and controlled the flow of information, as well as how their expression of scientific practice was related to their identity. Results provide further evidence for the diversity of people and practice involved in this domain of science and indicate that the magnitude and breadth of the public’s impact may be larger than previously projected. Certain types of messages were shown to be effective for different segments of the community, but news posts, essentially media outlet stories, were ineffective for generating any form of engagement. This study adds to our understanding of the important scientific contribution being made by members of the public as they interact with professional scientists and educators as peers in an open social media platform that supports a diverse and active community.

## Introduction

Social networks have a tremendous capacity for transforming science communication by making information, as well as the very practice of science, more accessible to all members of society [1]. For science domains that involve observations of the natural world, such as ornithology or paleontology, social networks can democratize science by affording the participation of all individuals, regardless of age, gender or background. Such forms of participation by non-professional members is recognized as citizen science, which has a long history of important contributions by members of the public [2]. For example, over a 74-year period from 1940–2014, 17% of the published studies related to monarch butterfly biology included data that was collected by citizen scientists [3].

While citizen science projects have a long and distinguished history, especially those that highlight location-specific biodiversity, little is known more generally about how the public interacts with scientific content and professional scientists on popular, open, digital platforms such as Twitter, Facebook, or Instagram. From an ecological perspective, scientific communities, which encompass citizen and professional scientists as well as informal learning collaborators such as museums, aquaria, and nature parks, are supported by a wide range of venues for communication that includes digital, print, and face-to-face forms. Each venue comprises a niche in an interconnected habitat [4], where the interaction within each niche is afforded by the environmental conditions [5]. For social media, prior research has shown these niches to be capable of supporting citizen science as a means for making important scientific contributions [6,7].

The field of paleontology involves an active scientific community that is engaged across multiple digital niches, including Facebook, Twitter and Instagram [8]. The community consists of such diverse participants as museums and their representatives, academic researchers, amateur fossil collectors (i.e. citizen scientists), paleontological artists, and commercial fossil dealers [9]. As such, it represents an ideal environment for examining the people, interactions, and flow of scientific information within social media niches, allowing us to more fully understand the conditions for and nature of participation in contemporary digital environments from the perspective of science as public participation.

Using interactions involving the four most popular hashtags for paleontology on Twitter over one month’s time, this study sought to address the following research questions: 1) Who are the members of this social world and how do they influence and control the flow of information? and 2) How is the expression of scientific practice within the social world related to self-identity with the domain of paleontology?

## Theoretical framework

This study is rooted in the symbolic interactionist tradition, a perspective that seeks to describe the development of people and the interrelated reciprocal relationship with transformation of social worlds that occur through communication [10]. A social world is an interconnected group of individuals who share purpose, ways of being and knowing as well as identity [11]. Social interaction creates structure and concurrently shapes people. Over time, social worlds develop through a semiotic web of social exchange, where communication serves as the means through which individual and group identity emerges and is maintained. Interaction in a social world can be captured as moments in time as a social network, a formative artifact that is indicative of a culture and trajectory, but not necessarily a predictable path or outcome. The existence of a social network implies a purpose for the group and for its members, a reason for interacting [12]. Furthermore, the group and its members can, and do, describe themselves within the network, using their account biographies as a narrative representation of their self-identity [13]. A connection among members (i.e. tie) within a social network signifies something previously shared and something potentially shared in the future. However, a community is more than its network structure as culture is made through the social interactions of the people who make up the community [11].

Dense networks, those with extensive internal connections and few outside connections are associated with trust, cooperation, mutual support and a willingness to communicate [14,15]. These networks offer the best potential for collective action because they provide needed support for individuals. However, such networks also come with expectations for behavior, ethics and responsibility for members. Reputation becomes very important in closed networks as it provides significant incentive for interaction.

## Related empirical studies

This paper advances our understanding of how science happens in open, publicly accessible digital spaces [16–18]. Current literature has addressed designed and organic social spaces as well as citizen science projects conducted online. While this research has added much to our understanding of how science is done online and how science communities form, little is known about how people participate in online science communities that form organically. Therefore, our goal is to provide a better understanding of how individuals participate in organically-formed online spaces where their interactions are based upon an interest in a science domain.

Citizen science is a popular method of participation in which people can become involved in science projects, engage with professional scientists in new ways, and contribute to research [19, 20]. Online citizen science projects, many of which are housed in clearinghouses such as SciStarter [21] and Zooniverse [22] exist in various science fields, including biology, environmental science, and earth science. In exemplar online citizen science projects, such as Galaxy Zoo, the designed digital space allows participants to make significant contributions to science and effectively connects the public with professional scientists [16, 17]. Educational research informs our understanding of participation in science within these designed online spaces where learners engage with authentic practice, such as middle school students collecting and analyzing biological data about horseshoe crabs [23] or discussing environmental science in online forums [24]. While these studies allow for the examination of scientific expertise and people's motivations for participation, they are limited in their sole focus on designed spaces.

Not all science enacted in digital environments is designed. Some digital environments encourage the development of communities where individuals can participate more freely, based upon their choice and agency. Allowing for a more organic environment, one where individuals can engage in communication and activities toward a common interest or goal, can lead to the formation of science communities of practice [25]. Some science communities of practice emerge in digital environments based upon achieving certain learning goals such as positively impacting biology teachers’ professional development experiences [26], as well as increasing students’ scientific writing skills [27]. Other communities have developed organically on social networks, including on Facebook, where birdwatchers can discuss birds that visit their feeders, identify species from photographs shared by other members, and communicate about bird biology [28]. In the same vein, Twitter has become a digital niche where science communities of practice can engage in conversation around different science topics of interest, such as identification of insect species [6].

Similarly, Twitter has been employed by scientists, journalists, and members of the public to discuss science. On Twitter, general discussions of science fall into the realm of practical advice or general rules of engagement for and with scientists [29]. For instance, Twitter use at scientific meetings by conference participants has been analyzed to determine active attendees, the content of communication, and the characteristics of posts [30–32]. Other Twitter-specific studies that focus on science have explored its capacity for networking [33] as well as the ways it can be used to communicate topical content to specialized audiences [34]. Twitter use in facilitating conversations about science itself trends towards species identification [6, 35]. Additionally, science journalists and communicators have used social media sites such as Twitter to effectively disseminate and communicate science-specific information [36, 37].

Online communication via social media is at an all-time high and not likely to diminish; determining communication topics and the people who discuss such topics is needed [38]. Within Twitter, organic communities that focus on science can be thought of as topic networks which highlight specific interest areas (e.g. health care, politics, or scientific disciplines) as opposed to attempting to understand the whole network [39]. These topic networks can be identified through keyword searching (e.g. “paleontology”) or through identifying hashtags associated with the topic (e.g. #paleontology). For a comprehensive overview of different topic networks and their structures, see the 2017 review done by Himelboim and colleagues [39]. In our view, Twitter topic networks are comprised of online, organic scientific communities, thus we sought to explore the social world that formed within one such topic network, which was created by the organic use of popular hashtags indicating the domain of paleontology.

## Methodology

This study involved an embedded mixed methods investigation of a social world in the form of a Twitter topic network focused on paleontology [40]. This social world was created by the relationships and interactions among participants, recognized here as members of the social world, through their use of the communication features and media conventions that were provided by the Twitter platform. For each member, the nature and quantity of social interactions and relationships, which result from their communication, are indicators of influence within the world [41]. In order to provide a better understanding of how the people involved with science communities interact in digital spaces, content analysis as well as Social Network Analysis (SNA) can be used. Networks can be analyzed to look for information about the network as a whole, or to look for individuals of interest, such as which person is most central or most connected [42]. While many studies have analyzed science communities in digital spaces, including Twitter, there is scant research on science communities on Twitter using popular non-conference/non-event related hashtags.

Our methods followed five distinct phases. The first phase, determining the most popular hashtags, was used to establish a valid method for procuring paleontology-specific tweets. Hashtagging (e.g., adding #paleontology to a post) is a user strategy for aggregating new content for a topic, which can increase the rate to which tweets are dispersed across a network [43]. With the website hashtagify.me, which provides a percentage-based system for reporting popularity of hashtags, we used an exhaustive search process involving key words (e.g., paleontology, fossil(s)), known hashtags for popular scientific meetings (e.g., #GSA2018, #2018SVP) and the reported lists of top correlated tags to our search terms. *#paleontology* was used as the seed to start the collection. For each hashtag in our search we reviewed the provided set of recent tweets to determine the degree to which they involved paleontology—the collection, preparation, curation, and study of fossils. If so, the hashtag was included. We then evaluated all of the top ten correlated tags and subsequent correlated tags in the same manner until all options had been evaluated. Some hashtags that were determined *a priori* were explored as potential additions to the list, however, most of these (e.g., #fossil, #dinosaur, #science) were determined to be not useful as their topics were far too broad or off topic. For example, we determined that #fossil is used to indicate the company that makes watches, whereas #fossils is indicative of paleontology. The hashtags that were determined to be appropriate and subsequently used for analysis were #FossilFriday (50.5% popularity,) #fossils (44.5% popularity), #paleontology (39.5% popularity), and #paleoart (35.3% popularity). Tweets related to paleontology at professional and academic conferences were considered (e.g., #2017SVP, #GSA2017, and #NSTA17), but were not included due to the narrow nature of the participants (i.e. predominantly professional scientists) and restricted context [44,45].

In phase two, we sampled Twitter using NodeXL [46] and Netlytic [47], two network extraction, analysis, and visualization software applications. These tools extract data from the public Twitter search API (application programming interface), providing a sample of tweets using a proprietary algorithm (i.e. a restricted sample, not the entire possible collection). These applications were used together because they interact with the API in different ways and thus address the potential for sampling bias, which can originate from a network snapshot. NodeXL performs an exhaustive single extraction based upon maximizing the API’s one-time limit, providing a depth of tweets from a narrow time point. Netlytic performs a series of timed extractions that provide less depth, but a greater breadth of tweets over time. Using both applications separately, then combining the results in NodeXL, we gathered tweets that used at least one of the four hashtags during a one-month period (August 26—September 26, 2017), resulting in a corpus of message data (N = 9,149). Data records included the text and images of each tweet, the relationship between the original author and anyone who passed the message along (i.e. re-tweet), responses that mentioned specific others (i.e. mentions) and the attributes of all involved accounts (e.g., biography, number of followers, etc.). Tweets are those messages that are created by a user, incorporating messaging elements such as URLs, mentions, and hashtags. Using Bruns and Stieglitz’s methodology [48] as a guide, we focused our analysis on tweets and retweets; mentions were included, but not the focus. These data were used in phase four to construct a social network [47]. All data collected complied with Twitter's terms of service, as laid out by Twitter's Developer Agreement and Policy, Section C entitled Respect Users' Control and Privacy. The research included Institutional Ethics Review and approval (UF-IRB201901701; NCSU-19060).

Phase three involved categorizing the members of the social network using a content analysis [49] of the account biographies as a narrative representation of their self-identity with paleontology [13]. This process differs from previous studies which categorized scientists based on external selection such as recruitment by the researchers [50], tweeting about science-specific journal articles [51], and using curated lists [52]. It is similar to methods used in [53], in which Twitter users were classified as students or professors based on their Twitter biographies. We expand upon this with a more naturalistic approach using the Paleontological Identity Taxonomy (PIT) [54], a tri-tiered hierarchical taxonomy (i.e. Structure, Category, Type) created by the authors for this purpose. The first tier, *Structure*, allows for classification at a coarse grain size, followed by the middle tier of *Category*, and lastly, the fine-grained classification of *Type*. Structure included the classifications of Individual, Organization, and Club/Group. Category encompassed Public, Scientist, Education and Outreach, and Commercial. Type varied by Category, with the Public breaking into three Types (i.e. Paleoartist, Amateur Paleontologist, and Interested Party); Scientist sub-divided into 10 Types, which were representative of scientific disciplines; Education and Outreach separated into 10 Types that represented different forms of education and outreach (e.g. K-12 teachers, informal education centers such as museums, and university members); and Commercial dividing into three Types (i.e. Experience, Resource, and Service). If biographies included a location, these metadata were also collected. Members were classified by the three authors, who individually coded all data then discussed any discrepancies to consensus during weekly meetings [55]. In two rounds of independent coding, interrater reliability was determined to have a significant level of agreement (Fleiss κ = 0.82, 0.78). The level of Category was used as the unit of analysis and our reporting indicates member categories as proper nouns (e.g., Scientist, Public) and since all member types are also categorically representative of categories we also report types as category-type with proper nouns (e.g., Scientist-Paleontology, Public-Interested Party).

Phase four involved a social network analysis of the tweets, retweets, and mentions as connections (i.e. edges) among the members of the social network (i.e. vertices), following the method described by Himelboim and colleagues [39]. The Twitter topic network was directed, meaning there was a clear origin of and direction to the relationships within the network: members of the network did not have to be “mutual” in order for a connection to exist. The flow of information can be determined in directed networks, as they show from where information originates (i.e. out-degree) and to whom it flows (i.e. out-degree). The network was visualized using the Harel-Koren fast multiscale graph and groups were determined with the Clauset-Newman-Moore clustering algorithm [56]. The following network and member-specific metrics were used as dependent variables: density (i.e. overall connectedness), eigenvector centrality (i.e. influence), betweenness centrality (i.e. control of information) and closeness centrality (i.e. individual connectedness). A one-way analysis of variance (ANOVA) with Tukey HSD post-hoc tests were used for between group comparisons. A Welch ANOVA with Games-Howell post hoc tests were used when the assumption of homogeneity of variances was not met.

The final phase entailed a content analysis to categorize the messages using the Paleontological Practice-based Post Type (P3T) framework [57], which allowed for identification and differentiation of scientific practice. With our focus on this specific type of communication, as occurring in multimodal and often cryptic ways due to limitations imposed by the platform, content analysis using human coders was chosen as the method for classifying posts over automated text analysis. With a restricted number of characters for a message, Twitter users make use of abbreviations, mentions, self-defined hashtags, images and video to expand the semantics of language in order to communicate [58]. Such content analysis has been utilized successfully in previous social media work, especially on Facebook, where researchers described four categories of posts based on message and intent, finding that messages could be described as motivational, invitational, informational, or investigational [59].

Within the current work, five unique post types were included in the codebook: Information, News, Opportunity, Research, and Off-Topic. Messages coded as *Information* contained general resources for paleontology, were disseminated posts of recent activity, links to blogs, or contained personal connections to paleontology. *News* posts were media outlet stories about paleontology that described the science for a lay audience. *Opportunity* posts were messages that indicated something in the field that community members or broader society could participate in. *Research* posts illustrated aspects of scientific research, including links to journal articles or fieldwork photos with scale bars and tools. Lastly, *Off-Topic* posts were messages not related to the science of paleontology, but instead were about a specific watch brand or political message about the fossil fuel industry. Post types were individually coded by a team of undergraduate interns as well as the first and second authors and then discussed to consensus in weekly meetings. Interrater reliability was determined to have a moderate level of agreement (Fleiss κ = 0.55). To better understand the flow of message types between groups within this social world, we used InfoMap, a community detection algorithm that maps information flow within the system [60]. Following the procedures of phase four, we collapsed members into groups based on the Clauset-Newman-Moore clustering algorithm in order to complete analysis at the group level.

## Results

**Fig 1:**
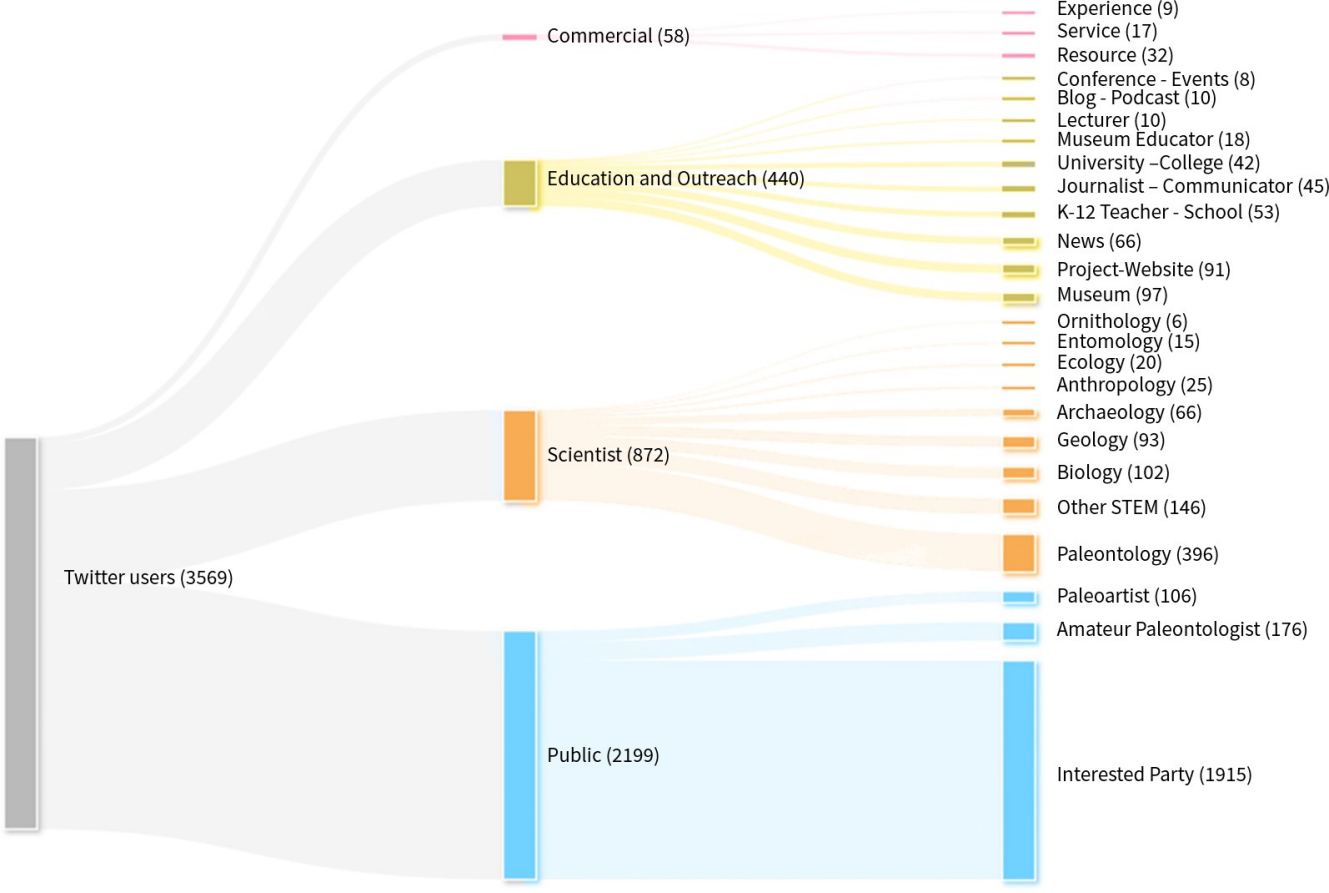
Quantities of members by classifications. Members are depicted in this diagram at the Categorical and Type levels of the PIT as members can be classified into such tiers regardless of Structure. At the Categorical level, Public made up the majority (61.6%, n = 2,199), followed by Scientists (24.4%, n = 872), Education and Outreach (12.3%, n = 440), and Commercial (1.6%, n = 58). At the Type level, Public-Interested Party made up the majority (53.6%, n = 1,915) whereas Scientist-Paleontologists made up just over 10 percent (11.1% n = 396).

The social world included 3,568 members (e.g., people, groups, institutions) and 9,149 connections (e.g., tweets, retweets, mentions). Structurally, 81 percent were Individuals (n = 2,913), 15 percent were Organizations (n = 540), and 3 percent were Clubs/Groups (n = 115). Regardless of Structure, 61 percent were members of the Public (n = 2,199), 24 percent were Scientists (n = 872), 12 percent were Education and Outreach (n = 440), and nearly 2 percent were Commercial (n = 58) (Fig 1). Within the classification of Type, Public-Interested Parties, those indicating minimal identity with paleontology, were the greatest majority of members (53%; n = 1,915) while Scientists-Paleontology, those indicating paleontological scientific work as a primary identity, accounted for just over ten percent (n = 396) of the social world. Public-Amateur Paleontologists, those indicating an interest in paleontology but not professional work, consisted of nearly five percent of the world (n = 176). Scientists indicating a STEM discipline not based in the physical or life sciences (e.g., social science, education) were four percent of the social world (n = 146). According to Chu, Gianvecchio, Wang, and Jajodia’s study [61] of more than 500,000 Twitter accounts, we can anticipate that roughly 53% of these entities were actual humans, 36% were cyborgs (i.e. humans using some form of software to automate and/or schedule their interactions) and 11% were bots (i.e. non-human, fully autonomous interactions generated by software).

Communication included 9,149 social interactions involving a range of types (e.g., tweet, reply, mention, retweet). Flow of information mainly involved the use of retweet (n = 7,235; 79%), where a member passed along an original message from another source to their collection of followers. Tweets, representing the introduction of new content (not necessarily original), accounted for nearly ten percent (n = 1,174) of the information flow (Table 1). Flow of information can be considered in terms of the content of the messages, including use of URLs and who is creating them [48]. Thus, we analyzed how different members within the network were contributing both tweets generally and specifically tweets with URLs. This analysis showed that the number of tweets produced by members of the Public was not proportional to the size of the group (.26 tweets per member). The Categories whose members were disseminating information at higher rates were Scientists (.38 tweets per member) and Education and Outreach (.46 tweets per member). Commercial members, which were proportionally the smallest percentage of the total, produced tweets at a rate of slightly over one tweet per member. We examined how members from different Categories were disseminating messages with URLs and found that the Categories of Public and Scientists’ tweets with URLs comprised of 74% of their total messages whereas Education and Outreach entities tweets with URLs accounted for 81% and Commercial members were even higher with close to 86%. These data highlight the ways that members influenced and controlled information within the network in that Commercial members included URLs at a higher rate, perhaps as a strategy to bring in new content and encourage consumer behavior. The number of tweets per entity contributed by Public and Scientist members highlight similarities in the ways that they contribute to the network.

**Table 1:** Tweets and use of URL by category.

	Tweets^a^	% of total	# of tweets per entity	Tweets with URLs^b^	% of total
Entity	*n*	%		*n*	%
Public	569	5%	0.26	420	74%
Scientist	333	3%	0.38	247	74%
Education and Outreach	203	2%	0.46	166	81.2
Commercial	69	<1%	1.19	59	85.5%

^a^N = 1,174

^b^N = 892

To explore the use of retweet as a form of influence, we assessed how this strategy was used by different Categories of members (Table 2). When examining retweet rates by Category, all members in all Categories retweeted at approximately the same rate, except for the Commercial Category, for whom the retweet rate was much lower at 0.7 percent. This shows that the information flow in the network took the form of retweets, especially by Public entities.

**Table 2:** Retweets by category.

	Retweets^a^	% of total connections	# of retweets per entity	# of retweets with URLs^b^	% of retweets with URLs
Public	4,731	65%	2.2	1,129	24%
Scientist	1,665	23%	1.9	363	2%
Education and Outreach	801	11%	1.8	171	2%
Commercial	38	<1%	0.7	17	45%

^a^N = 7,235

^b^N = 1,680

**Fig 2:**
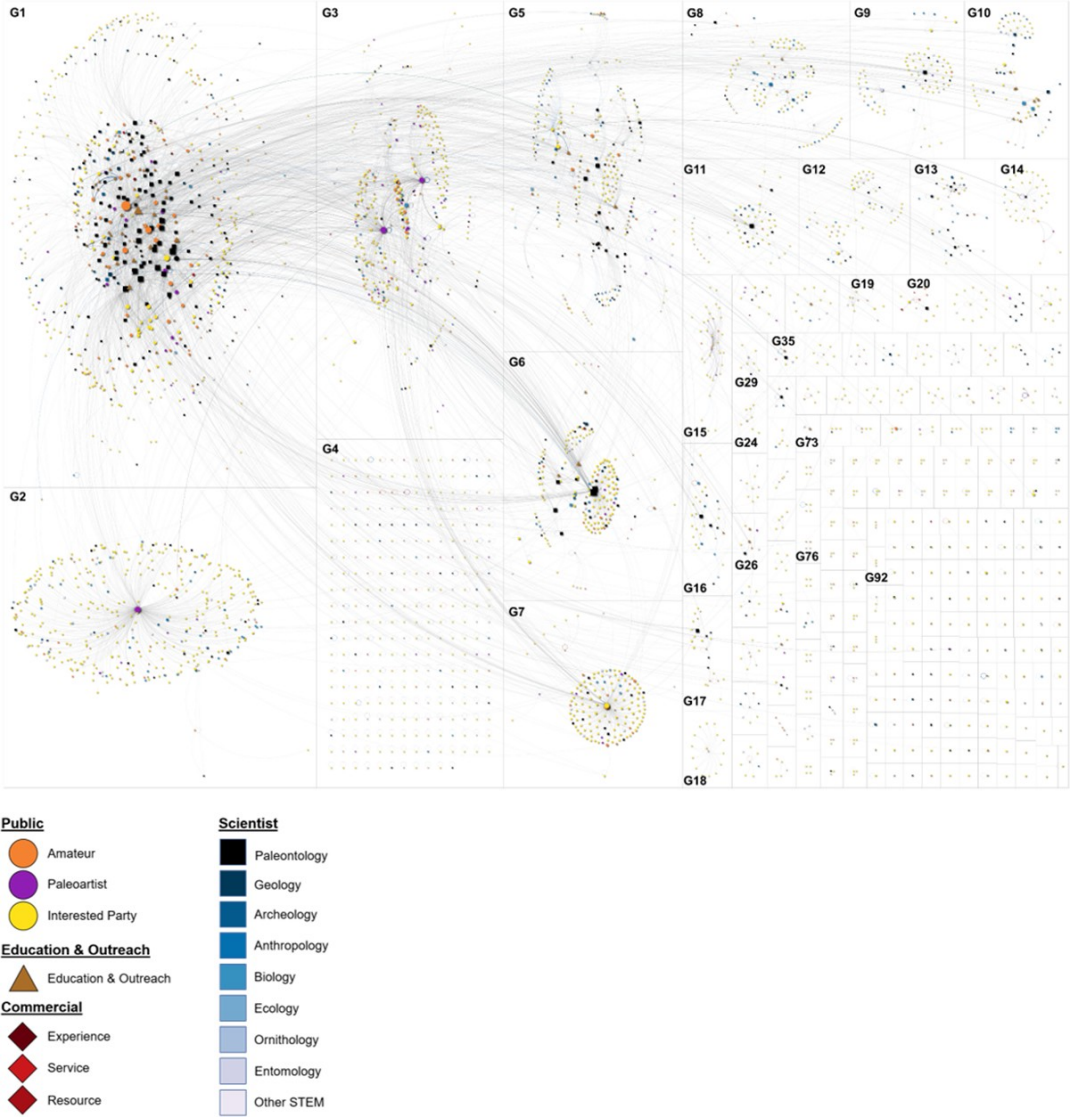
Network map following cluster analysis. Clusters are partitioned and labeled as groups (e.g., G1, G2, etc.). Members within groups are indicated by nodes, which are proportional in size to their degree of control (i.e. betweenness) and colorized by Category. Interactions are indicated as grey lines between nodes.

**Fig 3:**
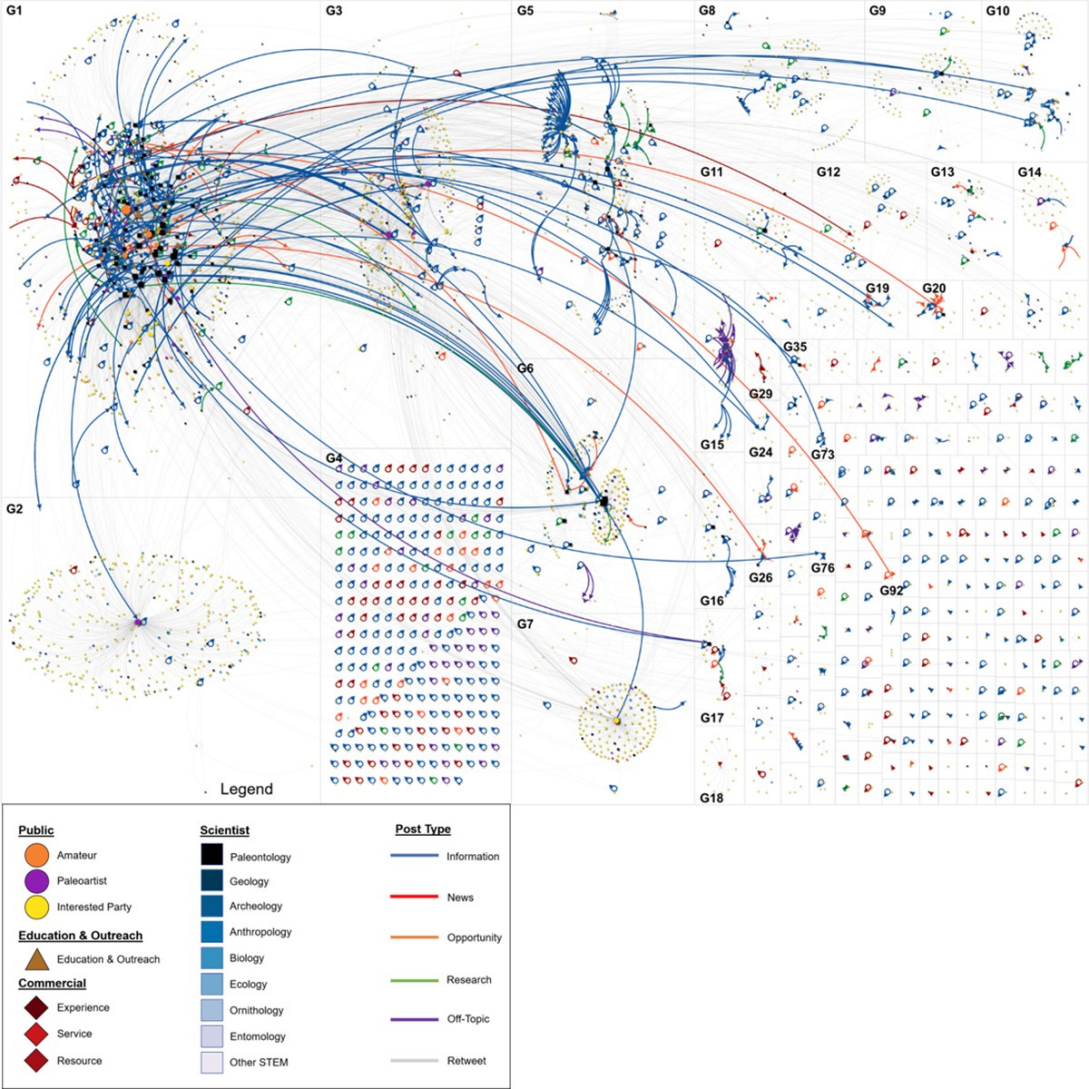
The flow of messages across groups within the social world. Members within groups are proportional in size to their degree of control (i.e. betweenness) and colorized by Category (Fig 2). New messages (i.e. tweets) are colorized by type.

We further characterized the flow of information and membership categories via describing the structure of the network. This social world was characterized as a low-density (ρ = 0.005) community cluster network (Table 3) [31]. Within this world, each one of the groups was a unique community that discussed different topics (Fig 2). Cluster analysis resulted in 238 groups, we discuss several of those groups here. The sociogram (Fig 3) shows many smaller groups, which mostly consisted of dyads—two entities communicating about a topic without that communication spreading to or including any other members of the social world (e.g., G92 in Fig 2). Other larger, more inclusive groups such as G1 consisted of community members who were able to connect to each other and to members of other groups. This contrasted with G4, in which members most often created posts that did not engage other members (i.e. self-loops). The discovery of the multitude of groups, including those groups which did not foment wider conversations, indicated a lack of density and that members were not deeply connected to each other or to topics mapped within the world.

**Table 3:** Network graph characteristics.

Graph Metric	Value
Graph Type	Directed
Vertices	3560
Total Edges	9149
Self-Loops	1198
Reciprocated Vertex Pair Ratio	0.0194
Reciprocated Edge Ratio	0.0381
Connected Components	467
Single-Vertex Connected Components	277
Maximum Vertices in a Connected Component	2686
Maximum Edges in a Connected Component	7985
Maximum Geodesic Distance (Diameter)	11
Average Geodesic Distance	3.91
Graph Density	0.000523
Modularity	0.528

Further analysis highlighted that groups had unique audiences, influential users, and sources of information. Most of the groups (e.g., G1-3, and G7 in Fig 2) were medium-sized with some of the most influential members at their center. The members who controlled the flow of information (i.e. were most central) were heterogeneous in their identities, representing different Structures, Categories, and Types. For example, a collection of approximately 20 Scientists-Paleontologists were central to G1, yet the three most influential contributors to this group were an Education and Outreach organization and two Public-Amateur Paleontologists. The hub and spoke structure of most groups indicated that central member(s) started different conversations, each with a different and unique audience. For example, the G2 cluster had a Public-Paleoartist at the center communicating information to their followers, in contrast to the Public-Interested Party at the center of the G7 cluster. In G2 and G3, the most influential members were Public-Paleoartists with largely Public audiences. G5 was diverse with a large number of members classified as Public, Scientist, or Education and Outreach. G6 was a large group that has a professional paleontology organization at the center. Influential members spanned different Structures, Categories, and Types, indicating that the flow of information was not limited; anyone could have substantial impact within the online, social world of paleontology.

A member’s position within the network indicated how they accessed information and subsequently, how their use of information influenced others in the social world (Table 4). One metric for determining how information is accessed and others are influenced in the social world is in-(out-)degree. Information originates from individuals with a high out-degree and flows to those with a high in-degree. The top ten members with high out-degree included seven members of the Public, two Scientists, and one Education and Outreach entity (Table 5). Those top ten members with highest in-degree included a more equal spread of members of the Public (n = 5) and Scientists (n = 4) and one Education and Outreach member (Table 6). Note that these top ten lists were exclusive to one another: there were no members who had both high in-degree and high out-degree. An additional metric to determine who within the network had the most control of information was betweenness centrality. It was determined that the Public, Scientists, and Education and Outreach members had equal control of information, with average betweenness increasing from the Public (M = 4,881, SD = 69,288), to Scientists (M = 7,158, SD = 27,284), to Education and Outreach (M = 5,957, SD = 60,186), but the differences between the groups was not statistically significant, F(2, 3,499) = 1.069, p = .343.

**Table 4:** Top 10 members by betweenness.

Rank	Structure	Category	Type	Betweenness Centrality	In-degree	Out-degree	Group
1	Individual	Public	Amateur	2,134,555	3	279	G1
2	Individual	Public	Paleoartist	2,041,292	442	1	G2
3	Individual	Education & Outreach	K-12 Teacher	1,149,719	0	217	G1
4	Individual	Public	Paleoartist	886,800	228	4	G3
5	Individual	Public	Paleoartist	594,612	171	3	G3
6	Individual	Public	Amateur	433,881	161	3	G7
7	Individual	Public	Amateur	400,580	0	86	G1
8	Organization	Scientist	Paleontology	390,139	158	2	G6
9	Individual	Public	Interested Party	303,451	157	0	G7
10	Individual	Scientist	Paleontology	289,811	64	2	G11

**Table 5:** Members with highest out-degree.

Rank	Structure	Category	Type	Out-degree	In-degree	Group
1	Individual	Public	Amateur	279	3	G1
2	Individual	Education and Outreach	K12 Teacher-School	217	0	G1
3	Individual	Public	Amateur	86	0	G1
4	Individual	Public	Interested Party	58	0	G1
5	Individual	Public	Amateur	54	13	G1
6	Individual	Public	Interested Party	48	0	G1
7	Individual	Scientist	Paleontology	44	1	G1
8	Individual	Public	Interested Party	42	18	G6
9	Individual	Public	Interested Party	42	0	G1
10	Individual	Scientist	Paleontology	39	18	G1

**Table 6:** Members with highest in-degree.

Rank	Structure	Category	Type	In-degree	Out-degree	Group
1	Individual	Public	Paleoartist	442	1	G1
2	Individual	Public	Paleoartist	228	4	G2
3	Individual	Public	Paleoartist	171	3	G3
4	Individual	Public	Amateur	161	3	G8
5	Organization	Scientist	Paleontology	158	2	G7
6	Individual	Public	Interested Party	157	0	G8
7	Organization	Scientist	Paleontology	111	1	G7
8	Organization	Education and Outreach	Museum-Science Center-Park	104	2	G1
9	Individual	Scientist	Paleontology	86	16	G1
10	Individual	Scientist	Paleontology	73	4	G1

Not all categories had the same degree of influence, F(2, 944) = 23.132, p < .0005. Influence, as measured by eigenvector centrality, was lowest for the Public (M = 2.19E-4, SD = 5.86E-4), higher for Education and Outreach (M = 3.62E-4, SD = 9.27E-4), and highest for Scientists (M = 4.04E-4, SD = 7.77E-4). However, the influence of Scientists was not found to be different from Education and Outreach members (p = .695), but both were significantly higher than that of the Public (p < .0005, p = .005). This implies that Scientists and Education and Outreach members were most highly connected, more likely to be connected to each other, and less likely to be connected to members of the Public. This finding has implications for information flow within this social world, indicating that messages from Scientists and Education and Outreach entities were likely to reach influential network members more effectively than those created by members of the Public.

Differences were also found in the average distance between entities of different categories, F(2, 1,041) = 17.291, p < .0005. Closeness centrality, a measure of the average shortest distance between members, was lowest for Scientists (M = 0.052, SD = 0.209), higher for the Public (M = 0.082, SD = 0.241), and highest for Education and Outreach (M = 0.144, SD = 0.309). Post hoc analysis revealed that each of these were significantly different from each other (p < .005). This finding indicates that Scientists were the most effective at disseminating their messages across the social world. Others have suggested [62] that higher closeness centralities, such as what was seen in Education and Outreach members, indicate dependency on others to disseminate information.

Comparing their closeness centralities, the Public and Scientists were determined to be equally close—suggesting mutual support and cooperation—but the Education and Outreach members were more than twice the distance away, F(4, 1365) = 4.69, p = .003—indicating a dependency in their relationship in terms of production and dissemination of information. Due to their limited numbers, Commercial members were excluded from these statistical analyses.

The expression of scientific practice varied by members’ self-identities. The Public made the largest contribution of tweets, accounting for 64% of the new content (Table 7). Scientists provided the largest number of Research posts, twice that of the Public, even though their total membership was one-third the size of that group. Commercial members only provided three total Research-specific tweets, but they provided a number of tweets in each of the other post types that were larger in proportion to their membership size than that of any other group.

**Table 7:** Message types by category.

	Information	Research	News	Opportunity	Off-Topic	Total
Public	647	50	65	64	129	955
Scientist	339	97	38	42	15	531
Education & Outreach	214	32	37	36	9	328
Commercial	34	3	8	32	20	97
Total	1,234	182	148	174	173	1,911

**Fig 4:**
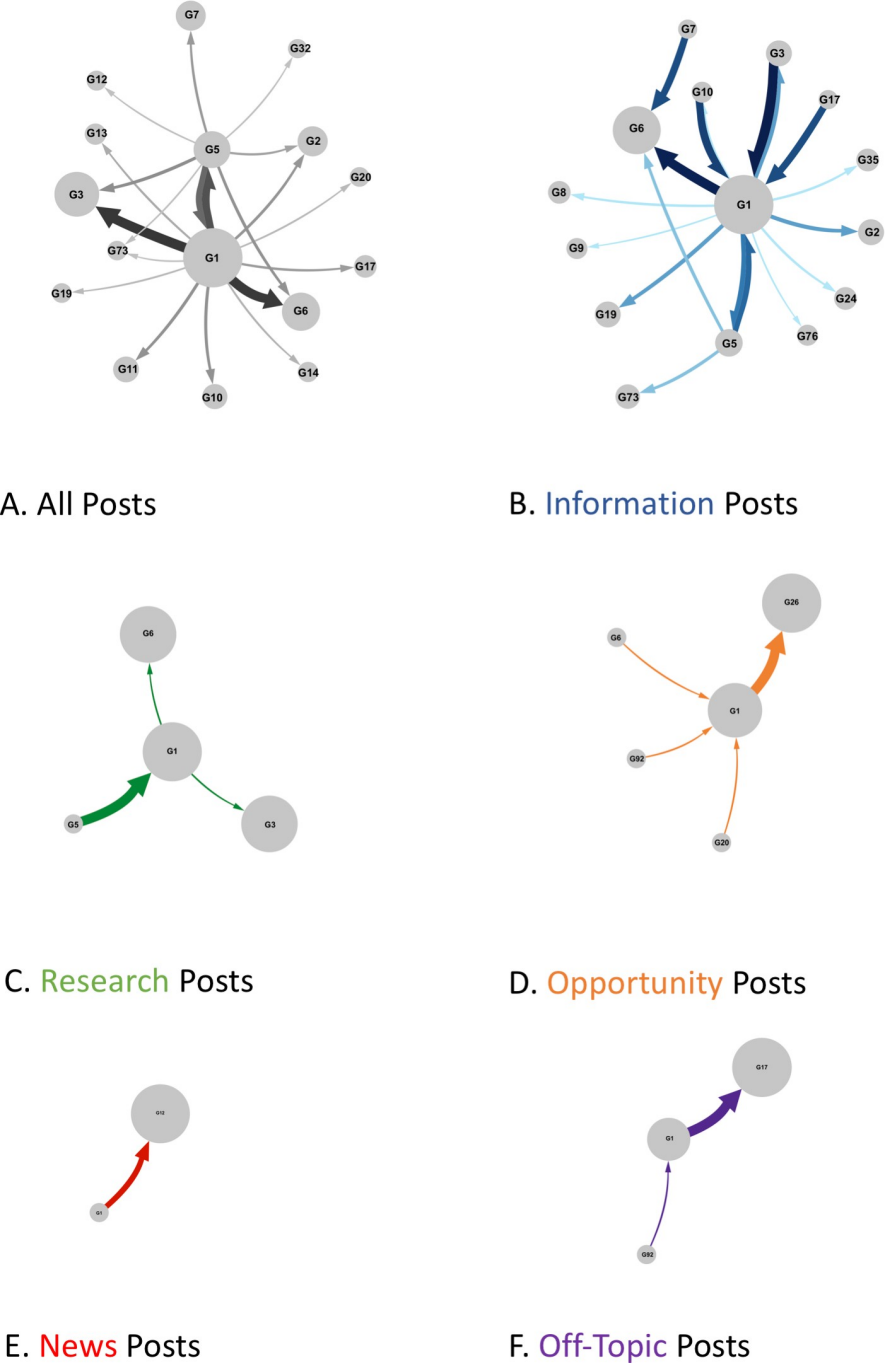
The flow of messages between groups by type. The link between the nodes represents the flow of messages between groups calculated using InfoMap. The node sizes are proportional to the number of members within each group.

We considered different post types identified by the P3T framework as ways that members of the social world self-identified with the domain of paleontology. As such, different post types were determined to be effective in producing interaction with different segments of the membership (Fig 3). Various post types were also determined to be effective in creating interactions between different groups within the social world (Fig 4).

The most successful, engaged with, and far-reaching expression of scientific practice were *Information* posts (Fig 4B). They also were the leading form of connection, consisting of 64.4% of all connections within the world. In addition, Information posts led to varied connections among all member types. When examining the network at the group level, we determined that Information posts occurred regardless of group and created the most connections between members of different groups (Fig 4B). Indeed, one specific group (G1) included a variety of member types who created and engaged with Information posts which subsequently spread to conversations within other groups. The distribution of Information posts regardless of group is an example of effective scientific social media messaging and engagement.

An additional form of effective scientific social media messaging was illustrated in the distribution and engagement of *Opportunity* posts across groups (Fig 4D). Opportunity posts, which consisted of 9.0% of all posts, were able to reach across groups, as exemplified by the number of posts from G1 that spread to G20, G26, and G92. Furthermore, Opportunity posts were created by different types of members and resulted in connections among all types. Thus, Information and Opportunity posts entailed the most effective messages, indicating that making personal connections to paleontology or being able to give others the ability to make their own personal connections to paleontology were paramount to members of this social world.

There were many instances where messages were ineffective at creating connections or generating further interest; or were only of interest to certain member types. For example, *Research* posts consisted of 9.7% of all connections and often created connections between various scientists as well as with Education and Outreach members (Fig 3). Research posts that were created by Scientists and Education and Outreach members within G1 often connected to other Scientists in G1 and G6. Additionally, there was one instance where an Amateur Paleontologist in G5 was directing research messages at an Education and Outreach Group in G1 (Fig 3). Research posts allowed for only certain members of the social world to connect—there were very few instances where the Public made or engaged with Research posts.

Additional ineffective messaging was found within the post type of *News* (Fig 4E). These posts were the least frequently utilized, at 7.7% of all posts, and were the most likely to result in self-loops (i.e. no one responding) and rarely connected members from different groups. On the infrequent occasions when members engaged with them, News posts created connections between all member types, aside from Commercial members. Lastly, *Off-Topic* posts, which consisted of 9.0% of all post types, also appeared within the world, with many of these posts focusing around one particular subject (Fig 4F) These Off-Topic posts co-opted a popular hashtag (#paleontology) in order to promote events that was unrelated to paleontology or to spread politically-charged messages. These reuses of a hashtag are examples of ineffective media messaging. Most often, News posts were created, yet not engaged with, while Research posts excluded a large segment of the social world (i.e. the Public). In summary, for the domain of paleontology, self-identity and expression infrequently centered on News and Research posts.

## Discussion

Our findings demonstrate that a diverse assortment of members have interest and impact on paleontological practice within a digital, social world that was created organically through people’s use of paleontology hashtags. This social world was primarily composed of the Public and Scientists. While it has been recognized that members of the public have significant impact on paleontological practice in the real-world [63]; these results are one of the first attempts to assess the magnitude and breadth of interest and impact on Twitter, regardless of status or level of expertise.

Within this world, members included those who identified as paleontologists as well as members with diverse interests and backgrounds. The world included Scientists-Anthropologists and Scientists-Archaeologists, as well as Public-Paleoartists who used the domain to spotlight their creations, and Commercial members who sold paleontology-specific goods and services. In another study of science on Twitter, Didegah, Mejlgaard and Sørensen [64] showed that a wide variety of members were involved in the communication of science research on Twitter and that both citizens and researchers played an important role. Such diversity implies not only a general interest in a scientific domain, but that Twitter can be accessible to a broad expanse of people.

The diversity of people who participated in this social world is likely responsible for its structure and low density. Members shared a common interest related to paleontology, but the cluster analysis indicated numerous conversations, each with different foci, resulting in a community cluster network [39]. This is illustrated by the composition of groups, and the most central member(s) in each group. The sub groups within this social world represented unique audiences that lacked deep connections. Structurally, this implies compartmentalized conversations that could provide evidence for homophily, filter bubbles, or echo chambers in which members with similar traits are highly connected [65,66,67]. Furthermore, such structures are indicative of information dissemination [68], a limited form of science communication that emphasizes distributing information over participatory conversation. Similar community cluster networks have been shown to form around topics such as #globalhealth [39], a broad general topic with a wide variety of subtopics, areas of foci, and issues such as infectious diseases, pollution, chronic non-communicable diseases, and climate change. These patterns are similar to those created for global news stories where the story topic defines the social world, but each news outlet has its own unique set of followers [65].

This social world was unique in its high percentage of retweets (79%) and low percentage of tweets containing URLs (9%). According to Bruns and Stieglitz’s Twitter hashtag typology [48], the ratio of retweets to tweets containing URLs can be used as a metric for how hashtags are being used. High retweet levels are more typically associated with tweets containing URLs. If these URLs are legitimate sources, such activity is indicative of broad dissemination or “passing along of situationally relevant information” (p. 176), such as in the case of breaking news events. If these URLs are malicious (i.e. spam), then this is an indicator of automated bots [61]. The lack of URLs for retweets in this social world suggests that the information content of the tweet was most likely from a human source. Furthermore, the content was retweeted because the members of the world viewed the content in a manner that was parallel to that of breaking news which did not require outside reference. Essentially, the retweets acted as first-person reports of potentially new scientific evidence. If this is the case, then such a pattern in the ratio of retweets to tweets with URLs could be a unique characteristic of a science-focused social world.

This study’s diverse social world had multiple types of influential people at the center of conversations. In many of the conversations, members of the Public were near the center or helped connect others to information within the network, which supports previous research indicating that members of the Public are key connectors and disseminators of paleontological information [63]. Such members act as bridges within the world, in that large amounts of information passed through them [62]. This suggests that those involved in the area of science communication should target their messages toward a public audience as that category of users has control over the spread of messages. In other research on Twitter, scientists, who disseminate and discuss their work, have been portrayed as the initiators of science-specific conversations [69]. This study supports such an assertion, where scientists are most influential, but also establishes a degree of influence for members of the public. Further, Scientist-Paleontologists were found to be influential within their sub groups, but not necessarily as influential overall. This indicates that scientists who want to use social media for scientific communication must be mindful of creating messages for the broader intellectual community as opposed to creating messages intended for other scientists. This finding supports the existing best practice communication strategies for to scientists [69], which were established with evidence from ecologists and evolutionary biologists [70], but had not been substantiated within the domain of paleontology.

Our analysis elucidated members’ self-identities within the domain of paleontology as expressions of scientific practice. We have shown that within a social world centered on paleontology, content can be delineated into four types (Research, Opportunity, News, and Information), as well as one unrelated type (Off-Topic posts). We described expressions of scientific practice via analyzing who, how and why people interacted with these post types. Previous work on social networks has revealed different categories of posts [59] and expressions of self-identity [28]. Studies that focus solely on descriptions of content are somewhat limited, as the content itself does not denote interaction. Bearing this caveat in mind, we do see some similarities in the descriptions of post types within this study and those previously described elsewhere.

Information posts—those indicating a personal experience or connection to paleontology—made up the majority of post types. This finding informs previous science communication research which indicated that narratively-driven stories or those that allowed for personal connections offer higher engagement with the content and the people who produce it [71]. Indeed, in research concerning health fields, researchers found that personal experiences and opinion sharing made up the majority of interactions [72]. Examining the flow of messages among members, we found that Information posts led to more connections among all member types. This suggests that members’ self-identity with the domain of paleontology foments connections as members can, and do, find ways to make others’ experiences relevant to their own.

These results show that Opportunity posts, those that indicated something in the field that community members or broader society could participate in, were effective for all members. Similarly, in their work on post types, Cardoso and colleagues [59] described invitational posts as “events inside or outside the community” (p. 239), which comprised nearly 50 percent of posts on their Facebook page. We see Cardoso and colleagues’ description of invitational posts as analogous to our description of Opportunity posts, and found that these post types created connections between and among all members. This shows that for a Twitter topic network centered on paleontology, members were interested in expressing practice through participation or highlighting ways for others to participate. We can extrapolate that the members of this social world are therefore open to broadening participation within the science of paleontology. However, this extrapolation must be tempered with the notion that although there were many connections between members, there were also many instances when messages failed to create any connections (i.e. creating self-loops).

Posts that were related to paleontology, such as Research posts, used scientific language to describe specific forms of practice. Research posts allowed conversations between Scientists to occur, as well as some degree of communication between Scientists and Education and Outreach members. However, Research-specific posts did not build conversation between members of the Public and other members, nor did Public members create large numbers of Research posts. This finding supports previous research from Didegah and colleagues [64], who found that there was little to no communication occurring in relation to tweeted scientific papers. For this world, a limited number of self-loops occurred where members would create a message that did not encourage conversation around the post’s topic. This finding aligns with previous work in other scientific fields, including dentistry [73] as well as biomedicine [74] in which scientists used Twitter to distribute journal articles, yet did so without encouraging discussion. Instead, members created tweets that were “devoid of original thought” [73] (p. 1). This critique indicates that research-specific tweets within scientific fields often fail to use basic science communication principles, including the use of dialogue to connect people to topics of interest [75]. For this world centered on the domain of paleontology, Research-based posts followed the same patterns.

News posts, essentially media outlet stories that were assumed to be intended for a general audience, also did not generate interaction and most often resulted in self-loops, a situation that we interpret as akin to talking to oneself. In the few instances where News posts generated engagement, Commercial members were not involved; only Scientists, Public, and Education and Outreach participated and did so minimally. This further illustrates the unproductive nature of News posts as they did not highlight scientific practice nor engender self-identity with the domain. This finding informs previous research by Bruns and colleagues [76], in which breaking news stories were often shared, but not necessarily commented on (i.e. retweeted with comments, or replied to). In some cases, sharing without commenting can be useful, as in the case of those who retweet news regarding natural disasters [48], however, within this social world, News stories about paleontology were not retweeted with such high frequencies. Furthermore, previous research indicates that messages crafted in ways that evoked emotional responses, had “perceived usefulness,” interest, or were positive in tone tended to be shared with higher frequencies [75] (p. 13647). Therefore, it could be that within this world, paleontology-specific news stories were not successful in meeting these criteria. Essentially, the nature of these stories was not perceived as paramount to the members of the social world.

Within all networks, occasional topically-irrelevant conversations can be expected as any social world exists within a larger social context. Within this topic network, Off-Topic posts were messages unrelated to the science of paleontology. These posts might have been capitalizing on the very nature of hashtags—enabling users to discover posts from “a very wide range of other contributors to the platform” [76] (p. 21). In essence, these specific posts comprised part of the network, but were too few to indicate a weak Twitter topic network.

Examined holistically, four of the five identified post types found in this paleontological social world entailed scientific practices that members freely chose to participate in. We relate the expression of scientific practice to self-identity with the domain as “…mak[ing] us able to cope with new situations in terms of past experience and gives us tools to plan for the future” [13] (p.16). Thus, the ways that members chose to participate in or contribute to this world indicated their involvement within the science of paleontology.

## Conclusion

This study adds to our understanding of the important scientific contribution being made by members of the public as they interact with professional scientists and educators as peers in one open digital social space within an ever-expansive habitat. Such scientific contribution is likely afforded by the nature of paleontology, with its basis in observations of the natural world as well as the range of ways that the science is being practiced, from collection, to digital curation and information dissemination to forms of artistic creation. However, it is also afforded by the open and more democratic capacity of the platform, which supports social interaction and collaboration based solely on interest. The Twitter platform is supporting the practice of citizen science in paleontology for a diverse group of participants in unique ways through interactions that illustrate the inherently social and humanistic way science is practiced. This practice includes how knowledge originates, is validated and curated, and how it translates through society based upon the needs and interests of all participants.

## References

[1] Brossard D, Scheufele DA. Science, New Media, and the Public. Science. 2013;339(6115):40–1. 10.1126/science.1232329 23288529

[2] Dickinson J, Bonney R. Citizen Science: Public participation in environmental research Ithaca, NY: Comstock Publishing Associates; 2012.

[3] Ries L, Oberhauser K. A Citizen Army for Science: Quantifying the Contributions of Citizen Scientists to our Understanding of Monarch Butterfly Biology. Bioscience. 2015 4 1;65(4):419–30.

[4] Wenger E, White N, Smith JD. Digital habitats: stewarding technology for communities Portland, OR: CPsquare; 2009.

[5] Gibson JJ. The ecological approach to visual perception Hillsdale, N.J.: Lawrence Erlbaum Associates; 1986.

[6] Daume S, Galaz V. “Anyone know what species this is?”—Twitter conversations as embryonic Citizen Science communities. PLoS ONE. 2016;11(3):e0151387 10.1371/journal.pone.0151387 26967526 PMC4788454

[7] Rossi RE. Using iNaturalist observations to detect disease in Red Mangroves (Rhizophora mangle). 2017 10 5; PeerJ Prepr 5:e3326v1

[8] MacFadden BJ, Lundgren LM, Crippen KJ, Dunckel BA, Ellis S. (2016). Amateur paleontological societies and fossil clubs, interactions with professional paleontologists, and the rise of 21st century social paleontology in the United States. Palaeontol Electron. 2016;19(2):1–19.

[9] Crippen KJ, Ellis S, Dunckel BA, Hendy AJW, MacFadden BJ. Seeking shared practice: A juxtaposition of the attributes and activities of organized fossil groups with those of professional paleontology. J Sci Educ Technol. 2016;25(5):731–746. 10.1007/s10956-016-9627-3

[10] Charon JM. Symbolic interactionism: an introduction, an interpretation, an integration Upper Saddle River N.J.: Pearson Education; 2009.

[11] Crossley N. Networks and complexity: directions for interactionist research? Symb Interact. 2011 12;33(3):341–63.

[12] Markham AN, Lindgren S. From Object to Flow: Network Sensibility, Symbolic Interactionism, and Social Media In: Johns MD, Chen S S, Terlip LA, editors. Symbolic Interaction and New Social Media. Emerald Group Publishing Limited; 2014 p. 7–41

[13] Sfard A, Prusak A. Telling identities: in search of an analytic tool for investigating learning as a culturally shaped activity. Educ Res 2005 5;34(4):14–22.

[14] Andrews KM, Delahaye BL. Influences on Knowledge processes In Organizational Learning: The Psychosocial Filter. J Manag Stud. 2002; 37(6): 797–810.

[15] Burt RS. Brokerage and closure: An introduction to social capital Oxford University Press; 2007

[16] Raddick MJ, Bracey G, Gay PL, Lintott CJ, Murray P, Schawinski K, et al Galaxy Zoo: Exploring the motivations of citizen science volunteers. Astron Educ Rev. 2010 12;9(1):1539–1515.

[17] Raddick MJ, Bracey G, Gay PL, Lintott CL, Cardamone C, Murray P, et al Galaxy Zoo: Motivations of citizen scientists. Astron Educ. 2013;12(1):1–27.

[18] Yocco V, Jones EC, Storksdieck M. Factors contributing to amateur astronomers’ involvement in education and public outreach. Astron Educ Rev. 2012 Dec;11(1):1–11.

[19] Doyle C, Li Y, Luczak-Roesch M, Anderson D, Glasson B, Boucher M, et al. What is online citizen science anyway? An educational perspective. arXiv:1805.00441v1 [Preprint]. 2018 [cited 2018 Dec 22]: [11 p.]. Available from: https://arxiv.org/abs/1805.00441v1

[20] Sturm U, Schade S, Ceccaroni L, Gold M, Kyba C, Claramunt B, et al Defining principles for mobile apps and platforms development in citizen science. RIO. 2018 1 4;4:e23394.

[21] Hoffman C, Cooper CB, Kennedy EB, Farooque M, Cavalier D. Scistarter 2.0: A digital platform to foster and study sustained engagement in citizen science In: Ceccaroni L, Piera J, editors. Analyzing the role of citizen science in modern research. IGI Global; 2017 p. 50–61.

[22] Simpson R, Page KR, De Roure D. Zooniverse: Observing the world’s largest citizen science platform. Proceedings of the 23rd International Conference on World Wide Web—WWW ‘14 Companion. New York, New York, USA: ACM Press; 2014. p. 1049–54.

[23] Hiller S, Kitsantas K. The Effect of a Horseshoe Crab Citizen Science Program on Middle School Student Science Performance and STEM Career Motivation. Sch Sci Math. 2014;114(6):302–11.

[24] Greenhow C, Gibbins T, Menzer MM. Re-thinking scientific literacy out-of-school: Arguing science issues in a niche Facebook application. Comput Human Behav. 2015 6 8;53:593–604.

[25] Lave J, Wenger E. Situated learning Cambridge, UK: Cambridge University Press; 1991.

[26] El-Hani CN, Greca IM. Compratica: A virtual community of practice for promoting biology teachers’ professional development in brazil. Res Sci Educ. 2013 8;43(4):1327–59.

[27] Kerlin SC, Carlsen WS, Kelly GJ, Goehring E. Global learning communities: A comparison of online domestic and international science class partnerships. J Sci Educ Technol. 2013 8;22(4):475–87.

[28] Liberatore A, Bowkett E, MacLeod CJ, Spurr E, Longnecker N. Social media as a platform for a citizen science community of practice. CSTP. 2018 3 20;3(1):1–3.

[29] Bik H. M., & Goldstein M. C. (2013). An introduction to social media for scientists. PLoS Biology, 11(4), e1001535 10.1371/journal.pbio.1001535 23630451 PMC3635859

[30] Bert F, Zeegers PD, Scaioli G. A social way to experience a scientific event: Twitter use at the 7th European Public Health Conference. Sc and J Public Health. 2016 3;44(2):130–3. 10.1177/1403494815612932 26511590

[31] Ekins S., & Perlstein E. O. (2014). Ten simple rules of live tweeting at scientific conferences. PLoS Computational Biology, 10(8), e1003789 10.1371/journal.pcbi.1003789 25144683 PMC4140634

[32] Bombaci S. P., Farr C. M., Gallo H. T., Mangan A. M., Stinson L. T., Kaushik M., & Pejchar L. (2016). Using Twitter to communicate conservation science from a professional conference. Conservation Biology, 30(1), 216–225. 10.1111/cobi.12570 26081769

[33] Van Noorden R. (2014). Scientists and the social network. Nature, 512, 126–129. 10.1038/512126a 25119221

[34] McHeyzer-Williams L. J., & McHeyzer-Williams M. G. (2016). Our year on twitter: science in #socialmedia. Trends in Immunology, 37(4), 260–265. 10.1016/j.it.2016.02.005 26979543

[35] Schuette S., Folk R. A., Cantley J. T., & Martine C. T. (2018). The hidden Heuchera: How science Twitter uncovered a globally imperiled species in Pennsylvania, USA. PhytoKeys, (96), 87–97. 10.3897/phytokeys.96.23667 29706786 PMC5915397

[36] Brown Jarreau P. (2015). Science bloggers’ self-perceived communication roles. Journal of Communication Management, 14(04). 10.22323/2.14040202

[37] Fahy D., & Nisbet M. C. (2011). The science journalist online: Shifting roles and emerging practices. Journalism, 12(7), 778–793. 10.1177/1464884911412697

[38] Perrin A. Social media usage: 2005–2015 Smith A, Page D, editors. Washington, D.C.: Pew Research Center; 2015.

[39] Himelboim I, Smith MA, Rainie L, Shneiderman B, Espina C. Classifying Twitter topic-networks using social network analysis. Soc Media + Soc. 2017 2 1;3(1):1–13.

[40] Creswell JW. Research Design: Qualitative, Quantitative and Mixed Methods Approaches. Los Angeles: Sage; 2014.

[41] Crossley N. The social world of the network: Combining qualitative and quantitative elements in social network analysis. Sociol. 2010 5(1):1–35.

[42] Brown ME, Ihli M, Hendrick O, Delgado-Arias S, Escobar VM, Griffith P. Social network and content analysis of the North American Carbon Program as a scientific community of practice. Soc Netw. 2016 11 6;44(1):226–37.

[43] Petrovic S, Osborne M, Lavrenko V. RT to win! Predicting message propagation in Twitter. In: Nicolov N, Shanahan JG, editors. Proceedings of the Fifth International AAAI Conference on Weblogs and Social Media. Barcelona, Catalonia, Spain: AAAI Press; 2011. p. 586–9.

[44] Risser HS, Waddell G. (2018). Beyond the backchannel: tweeting patterns after two educational conferences. EMI Educ Media Int. 2018 9 14;55(3):199–212.

[45] Xie Q, Luo T. (2018). Examining User Participation and Network Structure via an Analysis of a Twitter-Supported Conference Backchannel. J Educ Compu Res. 2018. Epub 2018 Aug 3.

[46] Smith M, Ceni A, Milic-Frayling N, Shneiderman B, Mendes Rodrigues E, Leskovec J, et al NodeXL: a free and open network overview, discovery and exploration add-in for Excel 2007/2010/2013/2016, http://nodexl.codeplex.com/ from the Social Media Research Foundation Social Media Research Foundation; 2010.

[47] Gruzd A, Paulin D, Haythornthwaite C. Analyzing social media and learning through content and social network analysis: A faceted methodological approach. JLA. 2016;3(3):46–71.

[48] Bruns A, Stieglitz S. Quantitative Approaches to Comparing Communication Patterns on Twitter. J Technol Hum Serv. 2012 7 1;30(3–4):160–85.

[49] Krippendorff K. Content analysis: An introduction to its methodology Los Angeles, Sage; 2012.

[50] Priem J, Costello KL. How and why scholars cite on Twitter. Proc Am Soc Info Sci Tech. 2010 2 3;47(1):1–4.

[51] Vainio J., & Holmberg K. (2017). Highly tweeted science articles: who tweets them? An analysis of Twitter user profile descriptions. Scientometrics, 112(1), 345–366. 10.1007/s11192-017-2368-0

[52] Ke Q, Ahn Y-Y, Sugimoto CR. A systematic identification and analysis of scientists on Twitter. PLoS ONE. 2017 4 11;12(4):e0175368 10.1371/journal.pone.0175368 28399145 PMC5388341

[53] Kimmons R., & Veletsianos G. (2016). Education scholars’ evolving uses of twitter as a conference backchannel and social commentary platform. British Journal of Educational Technology: Journal of the Council for Educational Technology, 47(3), 445–464. 10.1111/bjet.12428

[54] Lundgren LM, Crippen KJ, Bex, RT. Digging into the PIT: A new tool for characterizing the social paleontological community. Proceedings of E-Learn: World Conference on E-Learning in Corporate, Government, Healthcare, and Higher Education. Las Vegas, NV: Association for the Advancement of Computing in Education (AACE); 2018. p. 76–83

[55] Saldaña J. The coding manual for qualitative researchers Los Angeles: Sage; 2013.

[56] Clauset A, Newman MEJ, Moore C. Finding community structure in very large networks. Phys Rev E. 2004 12 6;70(6):066111. 10.1103/PhysRevE.70.066111 15697438

[57] Lundgren LM, Crippen KJ. Developing social paleontology: A case study implementing innovative social media applications In Remenyl D. (Ed.), The Social Media in Practice Excellence Awards 2017 at ECSM 2017: An Anthology of Case Histories. Reading, UK: Academic Conferences and Publishing International Limited (ACPIL); 2017 p. 11–26.

[58] Lacy S., Watson B. R., Riffe D., & Lovejoy J. (2015). Issues and best practices in content analysis. Journalism & Mass Communication Quarterly, 92(4), 791–811. 10.1177/1077699015607338

[59] Cardoso M, Warrick E, Golbeck J, Preece J. Motivational Impact of Facebook Posts on Environmental Communities. Proceedings of the 19th ACM Conference on Computer Supported Cooperative Work and Social Computing Companion. New York, NY: ACM; 2016. p. 237–40.

[60] Rosvall M., & Bergstrom C. T. (2008). Maps of random walks on complex networks reveal community structure. Proceedings of the National Academy of Sciences of the United States of America, 105(4), 1118–1123. 10.1073/pnas.0706851105 18216267 PMC2234100

[61] Chu Z, Gianvecchio S, Wang H, Jajodia S. Detecting Automation of Twitter Accounts: Are You a Human, Bot, or Cyborg? IEEE Trans Dependable Secure Comput, 2012 8 23;9(6):811–824.

[62] Hansen DL, Shneiderman B, Smith MA. Analyzing social media networks with NodeXL: Insights from a connected world Burlington, MA: Morgan Kaufmann; 2011.

[63] Catalani J. Contributions by amateur paleontologists in 21st century paleontology. Palaeontol Electron. 2014 5; 2(3E):4.

[64] Didegah F, Mejlgaard N, Sørensen MP. Investigating the quality of interactions and public engagement around scientific papers on Twitter. J Informetrics. 2018 8 14;12(3):960–71.

[65] Smith MA, Rainie L, Himelboim I, Shneiderman B. Mapping Twitter topic networks: From polarized crowds to community clusters Washington, D.C.: Pew Research Center; 2014 2.

[66] McPherson M, Smith-Lovin L, Cook JM. Birds of a Feather: Homophily in Social Networks. Annu Rev Sociol. 2001 8 1;27(1):415–44.

[67] Flaxman S., Goel S., & Rao J. M. (2016). Filter bubbles, echo chambers, and online news consumption. Public Opinion Quarterly, 80(S1), 298–320. 10.1093/poq/nfw006

[68] Tulu MM, Hou R, Younas T. Identifying influential nodes based on community structure to speed up the dissemination of information in complex network. IEEE Access. 2018 1 16;6:7390–401.

[69] McClain CR. Practices and promises of Facebook for science outreach: Becoming a “Nerd of Trust”. PLoS Biol. 2017 6 27;15(6):e2002020 10.1371/journal.pbio.2002020 28654674 PMC5486963

[70] Côté IM, Darling ES. Scientists on Twitter: Preaching to the choir or singing from the rooftops? FACETS. 2018 1 28;3(1):682–94.

[71] Dahlstrom MF. Using narratives and storytelling to communicate science with nonexpert audiences. Proc Natl Acad Sci USA. 2014 9 16;111 Suppl 4:13614–20 25225368 10.1073/pnas.1320645111 PMC4183170

[72] Lee NY, McElroy K. Online comments: The nature of comments on health journalism. Comput Human Behav. 2019 11 5;92(Mar):282–7.

[73] Robinson-Garcia N, Costas R, Isett K, Melkers J, Hicks D. The unbearable emptiness of tweeting-About journal articles. PLoS ONE. 2017 8 24;12(8):e0183551 10.1371/journal.pone.0183551 28837664 PMC5570264

[74] Haustein S, Peters I, Sugimoto CR, Thelwall M, Larivière V. Tweeting biomedicine: An analysis of tweets and citations in the biomedical literature. J Assoc Inf Sci Technol. 2014 11 26;65(4):656–69.

[75] Milkman KL, Berger J. The science of sharing and the sharing of science. Proc Natl Acad Sci USA. 2014 9 16;111 Suppl 4:13642–9. 25225360 10.1073/pnas.1317511111 PMC4183177

[76] Bruns A, Moon B, Paul A, Münch F. Towards a typology of hashtag publics: a large-scale comparative study of user engagement across trending topics. Comm Res Pract. 2016 1 2;2(1):20–46.

